# Case report: anesthesia management for emergency cesarean section in a patient with dwarfism

**DOI:** 10.1186/s12871-015-0048-2

**Published:** 2015-04-28

**Authors:** Xiaoxi Li, Hongjun Duan, Mingzhang Zuo

**Affiliations:** 1 Department of Anesthesiology, Beijing Cancer Hospital & Institute, Beijing, 100142 China; 2 Department of Anesthesiology, Beijing Hospital, Beijing, 100730 China

**Keywords:** Dwarfism, Cesarean section, Anesthesia

## Abstract

**Background:**

Dwarfism is characterized by short stature. Pregnancy in women with dwarfism is uncommon and cesarean section is generally indicated for delivery. Patients with dwarfism are high-risk population for both general and regional anesthesia, let alone in an emergency surgery.

**Case presentation:**

In this case report we present a 27-year-old Chinese puerpera with dwarfism who underwent emergency cesarean section under combined spinal and epidural anesthesia.

**Conclusion:**

It is an original case report, which provides instructive significance for anesthesia management especially combined spinal and epidural anesthesia in this rare condition. There was only one former article that reported a puerpera who underwent combined spinal and epidural anesthesia for a selective cesarean section.

## Background

Dwarfism, defined as an adult height under 145 cm in male or 135 cm in female, is a category of disorders with extreme global growth failure [1]. Fertility rates for women with dwarfism are generally low [2]. Puerperas with dwarfism usually require delivery by cesarean section because of contracted pelvis and cephalopelvic disproportion. However, anesthesia management in dwarfism is challenging, for it is often complicated by conditions such as deformed spine, limited neck mobility, and narrowed pharynx, leading to high-risk in both general anesthesia and regional anesthesia. Here, we present you a successful case of the anesthetic management in a puerpera with dwarfism undergoing cesarean section.

## Case presentation

A 27-year-old primipara with dwarfism at 38 weeks gestation was presented to the OR for an emergency cesarean section. She had typical features of disproportionate dwarfism, with short limbs and lumber lordosis. She was 120 cm tall, largely made up of her head and body trunk, and weighed 63.5 kg. She had normal intelligence. Coexistent disorder in her mother suggests she may have a positive family history. A magnetic resonance imaging (MRI) scan of her spine taken before pregnancy showed a spinal deformity (slight lumbar lordosis), but no cord compressions. The level of termination of the spinal cord was at the L1-2 interspace. No pre-existing cardiorespiratory dysfunction was found by pre-operational assessment. Other laboratory results were normal. On spine examination, we found that her lumber intervertebral spaces were palpable, but her thoracic intervertebral spaces were not. Obstetrical ultrasound scan revealed that the fetus was normally developed and in a transverse lie. After discussion with the obstetrician and the patient, we planned to administer combined spinal epidural anesthesia with a backup plan of general anesthesia.

Routine non-invasive monitoring was established, including non-invasive blood pressure (NBP), heart rate (HR), pulse oximetry (SpO_2_), and electrocardiography (ECG). Her baseline blood pressure (BP) and HR were 138/63 mmHg and 102/min, respectively. Her SpO_2_ was 99% while breathing room air and ECG results showed normal sinus rhythm. After intravenous access was obtained, the patient was placed in the left lateral decubitus position. 3 ml of 1% lidocaine was used for local infiltration anesthesia at L2-3 interspace. The epidural space was located with a 17G Tuohy needle at the first attempt. Then a 25-gauge pencil-point spinal needle was inserted via the Tuohy needle. After cerebrospinal fluid was detected, 1.4 ml 0.5% hypobaric bupivacaine was given into the subarachnoid cavity, with the speed of 6 sec/ml. Finally, an epidural catheter was placed into the epidural space. The process was uneventful and patient restored her supine position with the operating table slightly leaned to the left. Almost instantly, the patient’s BP declined to 72/46 mmHg and HR to 70/min. 10 mg Ephedrine was immediately administered intravenously. At this stage, the block height reached T4 bilaterally. After 0.2 mg Metaradrine was further administered, her BP increased to 110/62 mmHg and HR to 90/min. 5 minutes later, the block height remained at T4 and surgery was started. With intermittent administration of Ephedrine and Metaradrine, the patient’s BP was remained between 90-100/55-62 mmHg. There were no other complaints from the patient except for a slight feeling of breathless, which was relieved after a male baby was delivered. Subsequently, the patient’s BP gradually increased to 110-130/57-73 mmHg and remained stable during operation. The surgery lasted 30 minutes. The epidural catheter was removed and the patient was transferred to the post-operative anesthetic care unit for further observation. She was sent back to the obstetric unit 1 hour later when the block height declined to T6. The Apgar scores for the newborn infant were 10 at 1 and 5 minutes respectively. No peri-operative or anesthetic complications were observed. The patient was discharged home three days later.

## Discussion

People with dwarfism have generally been devided into two categories: short-statured individuals with approximately normal anthropometric proportions and those with disproportionate development, characterized by short limbs or short trunks, which are deformed in many cases. Among these, achondroplastic dwarfism is the most common type of human dwarfism with a genetic disorder of bone growth. It occurs in approximately 1 in 15,000 to 1 in 40,000 live births. Achondroplasia dwarfism can be inherited in an autosomal dominant pattern. However, 80% achondroplasia occurs with no family history. The characteristic features of achondroplasia include typical facial features, disproportionate short stature, and an exaggerated lumbar lordosis. Severe spinal deformity may also lead to cord compression. In our case, according to her appearance, lack of dysgnosia, and hereditary pattern, we consider the possibility that she had achondroplasia, although the cause for her growth deficiency is unknown.

The anesthetic management of the patient with dwarfism for cesarean section presents significant challenge for anesthesiologists, not only due to the pathophysiological diversifications of various forms of dwarfism and relatively rare pregnancy and labor in women with dwarfism, but also due to poor bony landmarks, spinal deformity and no standard guidelines for anesthetic management. The most popular anesthetic management for cesarean section is combined spinal epidural anesthesia. However, it may be technically difficult to perform in the patients with significant physical abnormalities, such as severe lumber lordosis, spinal deformity, and potential cord compression. In addition, the risk of intrathecal anesthesia for pregnant patients with dwarfism is high due to a lack of X-ray examination, which is not usually indicated for pregnant patients. Therefore the severity of spinal deformity is often difficult to be evaluated. In this case, the patient had minor lumber lordosis, and she’s never had symptoms of cord compression before. On examination, we were able to palpate the intervertebral spaces in the patient’s lumbar region. Therefore, we decided to administer combined spinal epidural anesthesia. Noticeably, the patient has small features but normal body trunk, and the volume of subarachnoid and epidural spaces are uncertain. For patient like her, the most appropriate type and dose of local anesthetics are unclear. Considering the length of her body trunk is very close to that of normal adult, we speculated that we should not calculate the dose of anesthetics based only on her height. After careful consideration and discussion, we decided to give her a relatively lower dose (1.4 ml) of 0.5% hypobaric bupivacaine rather than routine dose (1.8-1.9 ml) for spinal anesthesia. We also prepared 2% lidocaine for epidural anesthesia if needed. The patient achieved a block height at T4 almost immediately after the anesthesia procedure was accomplished and the surgery was uneventful.

When we searched the literatures for the reference dosages of anesthetics administered to the patients with dwarfisms afterwards, we noticed that the dosage we used was consistent with that reported by Samra, who used the minimum effective dose (providing effective spinal block in 95% of the women undergoing cesarean section) of intrathecal bupivacaine (0.06 mg/cm height) in a puerpera with achondroplasia, and achieved satisfying block height [3]. Therefore, we recommend that the dosage of local anesthetics used for spinal anesthesia in these patients should be no more than 0.06 mg/cm height to avoid an exaggerated block height leading to unstable hemodynamic. An epidural catheter should always be placed whenever possible in case of individual variation.

So far, the reports regarding regional anesthesia in patients with dwarfism undergoing cesarean section are scarce. According to the previous reports including ours, we believe local anesthesia is optional for cesarean section [4-6]. Nevertheless, anesthesia poses a significant risk to patients with dwarfism, which should be recognized by all anesthesiologists. Preoperative assessments should be carefully made and the contraindications such as severe spinal deformity or cord compression should be ruled out. Patients’ X-rays, or MRI examination taken before pregnancy is helpful in preoperative evaluation. Local anesthesia (spinal or epidural anesthesia) should be carefully planned. Close monitoring is important. Another option is to perform general anesthesia [2], in which case difficult airway places great risk due to the conditions such as big head and tongue, limited head extension, cervical spine instability and relatively undeveloped pharynx. Therefore, patients should be carefully evaluated to exclude difficult airway using indicators such as Mallampati classification, thyromental distance, sternomental distance. Different techniques for management of difficult airway should be prepared in advance. For patients with high risk of difficult airway, awake intubation should be considered. Postoperative recovery may be affected when impaired cardiopulmonary function exists.

## Conclusions

In our case report, we presented a successful anesthesia management of a puerpera with dwarfism who underwent emergency cesarean section. Patients with dwarfism are at high-risk for both general and regional anesthesia, especially in emergency surgeries. However, with careful examination and evaluation, intraspinal anesthesia were successfully planned and performed in our case. It provides instructive significance for anesthesia management in this rare condition.

## Consent

Written informed consent was obtained from the patient for publication of this Case report. A copy of the written consent is available for review by the Editor of this journal.

## Abbreviations

MRI: Magnetic resonance imaging
NBP: Non-invasive blood pressure
HR: Heart rate
SpO_2_: Pulse oximetry
ECG: Electrocardiography
BP: Blood pressure
